# Clinical outcomes in patients treated for coronary in-stent restenosis with drug-eluting balloons: Impact of high platelet reactivity

**DOI:** 10.1371/journal.pone.0188493

**Published:** 2017-12-07

**Authors:** Adrienn Tornyos, Dániel Aradi, Iván G. Horváth, Attila Kónyi, Balázs Magyari, Tünde Pintér, András Vorobcsuk, Dániel Tornyos, András Komócsi

**Affiliations:** 1 Department of Interventional Cardiology, Heart Institute, University of Pécs, Pécs, Hungary; 2 Heart Centre Balatonfüred and Heart and Vascular Centre, Semmelweis University, Budapest, Hungary; University of Tampere, FINLAND

## Abstract

**Background:**

The impact of high platelet reactivity (HPR) on clinical outcomes after elective percutaneous coronary interventions (PCI) with drug-eluting balloons (DEB) due to in-stent restenosis (ISR) is unknown.

**Objective:**

We sought to evaluate the prognostic importance of HPR together with conventional risk factors in patients treated with DEB.

**Methods:**

Patients treated with DEB due to ISR were enrolled in a single-centre, prospective registry between October 2009 and March 2015. Only patients with recent myocardial infarction (MI) received prasugrel, others were treated with clopidogrel. HPR was defined as an ADP-test >46U with the Multiplate assay and no adjustments were done based on results. The primary endpoint of the study was a composite of cardiovascular mortality, MI, any revascularization or stroke during one-year follow-up.

**Results:**

194 stable angina patients were recruited of whom 90% were treated with clopidogrel. Clinical characteristics and procedural data were available for all patients; while platelet function testing was performed in 152 subjects of whom 32 (21%) had HPR. Patients with HPR had a higher risk for the primary endpoint (HR: 2.45; CI: 1.01–5.92; p = 0.03). The difference was primarily driven by a higher risk for revascularization and MI. According to the multivariate analysis, HPR remained a significant, independent predictor of the primary endpoint (HR: 2.88; CI: 1.02–8.14; p = 0.04), while total DEB length and statin treatment were other independent correlates of the primary outcome.

**Conclusion:**

HPR was found to be an independent predictor of repeat revascularization and MI among elective patients with ISR undergoing PCI with DEB.

## Introduction

Coronary in-stent restenosis (ISR) is a common complication with bare metal stents and is still a limitation of concern with current-generation drug-eluting stents.[1] In the last few years, drug-eluting balloon (DEB) dilatation has emerged as a therapeutic alternative to drug eluting stent (DES) implantation for percutaneous treatment of ISR. DEB may offer a benefit to treat ISR without repeat implantation of a metal layer into the restenotic mass, that itself, with the polymer on its surface is a key driver of the adverse process. [2] Several randomized studies have demonstrated the safety and efficacy of this technology. [3] [4] Moreover, a meta-analysis including more than 1400 patients demonstrated that DEB was clinically non-inferior to DES in the treatment of ISR in different clinical scenarios. [2]

After DEB percutaneous coronary intervention (PCI), patients receive double antiplatelet therapy (DAPT) for a certain timeframe but the optimal intensity and duration is not clearly defined. High residual platelet reactivity (HPR) in patients on clopidogrel has been demonstrated to be a strong and independent predictor of recurrent ischemic events and mortality in patients after coronary stent implantation. [5]

However, the relevance of HPR in the setting of ISR and DEB dilation is unknown. A prior study with optical coherence tomography (OCT) demonstrated uncovered or malapposed stent struts and intimal dissections in DEB-dilated segments [6] that may provide a prothrombotic surface after balloon dilation. As healing may be delayed consequent to paclitaxel treatment, this may have potential implications regarding the efficacy of antiplatelet therapy. The objective of our study was to evaluate the impact of HPR together with patient-related and procedural characteristics on clinical outcomes in patients with ISR treated with DEB.

## Methods

### Population

Starting on October 1, 2009, patients treated with DEB for ISR were enrolled in a single centre prospective registry in the Heart Institute, University of Pécs. There were no exclusion criteria; this was an all-comer registry. The registry adhered to the tenets of the most recent revision of the Declaration of Helsinki. The institutional ethical board (Regional Research Ethics Committee, University of Pécs, Clinical Centre) reviewed the protocol and approved this study under the protocol number 3551/2009. All included subjects have been properly instructed and have given written informed consent.

### Registered data

Data were collected prospectively from dedicated hospital records. Follow-up data were obtained at clinical presentations and at a telephone visit scheduled at 12 months after the index DEB PCI. Detailed procedural parameters of the intervention as well as risk factors, demographic data, medication information and laboratory parameters were also registered (Table 1).

**Table 1:** Baseline characteristics of the patient population.

Baseline characteristics	Entire patient population (n = 194)	HPR (n = 32)	no HPR (n = 120)	p¶
Clinical characteristics				
• Age	59.7 (31.4–85.7)	57.7 (37.9–72.2)	59.6 (31.4–85.7)	0.15
• Male	118 (60.6)	23 (71.9)	67 (55.8)	0.11
• Smoking	47 (24.1)	10 (31.3)	25 (20.8)	0.24
• Hypertension	173 (88.7)	27 (84.4)	112 (93.3)	0.14
• Diabetes mellitus	48 (24.6)	11 (34.4)	29 (24.2)	0.26
• Statin treatment (dyslipidaemia)	95 (48.7)	15 (46.9)	60 (50.0)	0.84
• Prior MI	135 (69.2)	23 (71.9)	85 (70.8)	1.00
• Prior CABG	36 (18.5)	3 (9.4)	26 (21.7)	0.14
• High platelet reactivity	32 (16.4)			
Prior PCI procedure				
• Indication				0.93
• STEMI	63 (32.5)	11 (34.4)	36 (30.0)	
• NSTEMI	17 (8.8)	3 (9.4)	9 (7.5)	
• Unstable angina	26 (13.4)	5 (15.7)	19 (15.8)	
• Stable angina	82 (42.3)	12 (37.5)	51 (42.5)	
• Target vessel				0.14
• LAD	76 (39.2)	10 (31.3)	51 (42.5)	
• RCA	86 (44.3)	20 (62.5)	50 (41.7)	
• CX	24 (12.4)	2 (6.3)	12 (10.0)	
• Graft	8 (4.1)	0 (0.0)	7 (5.8)	
• Total stent length, mm	30 (8–138)	31 (13–101)	30 (8–138)	0.88
• Stent count/patient	2 (1–7)	2 (1–5)	2 (1–7)	0.64
• Drug eluting stent	51 (26.2)	4 (12.5)	37(30.8)	0.04
DEB procedure				
• Type of DEB				0.04
• SeQuent Please	58 (29.9)	4 (12.5)	43 (35.8)	
• Invatec In-Pact.	70 (36.1)	16 (50.0)	45 (37.5)	
• Protege	39 (20.1)	10 (31.3)	22 (18.3)	
• Pantera Lux	24 (12.4)	1 (3.1)	9 (7.5)	
• Total DEB length, mm	25.5 (12–90)	23 (12–60)	20 (12–60)	0.78
• Total DEB length ≥ 22.5, mm	103 (52.8)	16 (50.0)	59 (49.2)	1.00
• Largest DEB diameter, mm	3 (2.5–5)	3.5 (2.5–4)	3 (2.5–5)	0.19
• DEB count/patient	1 (1–4)	1 (1–2)	1 (1–2)	0.71
• Predilatation	169 (86.7)	28 (87.5)	107 (89.2)	0.75
• ADP-test, U	28 (2–91)			
• ADP-test ≥ 52.5, U	23 (11.8)			
• ADP-test ≥ 63.5, U	15 (7.7)			
• Type 4 MI troponin	13 (6.7)	4 (12.5)	6 (5.0)	0.21
• Complication	31 (15.9)	6 (18.8)	22 (18.3)	1.00
Laboratory findings				
• Red blood cell distribution width	15.2 ± 1.9	14.3 (13.6–18.2)	15.1 (12.5–17.1)	0.77
• Leukocyte count, g/l	7.9 (4.5–13.7)	8.5 ± 3.0	8.5 ± 1.9	0.98
• Platelet count, g/l	251 ± 60	286.8 ± 83.5	248.7 ± 57.2	0.33
• Mean platelet volume	8.7 ± 1.1	8.3 ± 1.2	8.8 ± 1.1	0.33
• C-reactive protein, mg/l	2.2 (0.6–23.6)	12.1 ± 11.0	2.2 ± 1.6	0.26
Discharge medication				
• Clopidogrel	175 (90.2)	25 (78.1)	111(92.5)	0.04
• Prasugrel	17 (8.8)†	7 (21.9)	8 (6.7)	0.02
• ACE-I	139 (71.3)	23 (71.9)	84 (70.0)	1.00
• ARB	39 (20.0)	9 (28.1)	23 (19.2)	0.32
• Beta-blocker	155 (79.5)	25 (78.1)	96 (80.0)	0.81
• Calcium channel blocker	65 (33.3)	11 (34.4)	40 (33.3)	1.00
• Allopurinol	17 (8.7)	0 (0)	9 (7.5)	0.21
• PPI	158 (81.0)	28 (87.5)	95 (79.2)	0.45
• Antacid	37 (18.9)	5 (15.6)	25 (20.8)	0.62

Values are mean ± SD, n (%), or median (interquartile range).

¶ Comparison between HPR vs. no HPR patients.

† 2 (1%) patients received ticlopidine therapy.

HPR = high platelet reactivity; MI = myocardial infarction; CABG = coronary artery bypass graft; PCI = percutaneous coronary intervention; STEMI = ST-segment elevation myocardial infarction; NSTEMI = non–ST-segment elevation myocardial infarction; LAD = left anterior descending; RCA = right coronary artery; Cx = circumflex artery; DEB = drug eluting balloon; ADP = adenosine diphosphate; ACE-I = angiotensin-converting enzyme inhibitor; ARB = angiotensin receptor blocker; PPI = proton pump inhibitor

### Percutaneous coronary intervention and antiplatelet therapy

The selection of technique and revascularization strategy was at the discretion of the operators, including the choice of vascular access, type and number of DEB and need for pre- or post-dilatation or bailout stenting. Antiplatelet treatment was given according to the actual European guidelines of myocardial revascularization and treatment of stable angina.[7] All patients received 100 mg of aspirin, and clopidogrel was the choice from oral P2Y12-inhibitors. Only a small group of patients with prior acute myocardial infarction (AMI) within a year were treated with prasugrel that was continued regardless of the platelet function testing. Patients on clopidogrel continued treatment with an optional loading dose at the time of PCI decided by the operator. Patients without chronic P2Y12-inhibitors were treated with a 300/600 mg loading dose of clopidogrel, followed by a maintenance dose of 75 mg/day. Dual antiplatelet therapy was proposed to maintain during 12 months after DEB PCI.

### Platelet function testing

Antecubital venous blood samples were collected using a sterile 21-gauge needle into hirudin coated vacuum tubes (Becton and Dickinson, Munich, Germany) without stasis. Platelet function testing was performed with the Multiplate analyser (Roche Diagnostics, Rotkreuz, Switzerland), at least 6 hours after PCI. HPR was defined according to the consensus cut-off, which was an adenosine diphosphate (ADP)-test value greater than 46 U. [8] Importantly, results of platelet function testing did not lead to treatment corrections regarding P2Y12-inhibitor treatment.

### Endpoints and follow-up

Patients were followed for one year after DEB intervention. The primary endpoint of the study was the occurrence of major adverse cardiac events (MACE) defined as the composite of cardiovascular (CV) mortality, any revascularization, myocardial infarction (MI) or stroke/transient ischemic attack (TIA). Secondary endpoints included the individual elements of the composite endpoint.

Any revascularization included percutaneous or surgical interventions of the coronary arteries after the DEB PCI. MI was defined according to the universal definition.[9] Type 4 periprocedural MI was not considered as an endpoint. Stroke and TIA was defined according to American Heart Association/American Stroke Association definition.[10] [11]

### Statistical analysis

Continuous variables with normal distribution are presented as mean ± standard deviation (SD), whereas non-normally distributed variables are presented as median and interquartile range. Categorical variables are expressed as frequencies and percentages. Differences between groups were assessed with the Fisher’s exact test or chi-square test, as appropriate for categorical variables. Unpaired t tests were used for comparisons of normally distributed continuous variables, whereas non-normally distributed variables were compared using the Mann-Whitney U test. Hazard ratios (HR) and 95% confidence intervals (CI) were calculated for occurrence of clinical endpoints at follow-up. Cox regression and Kaplan-Meier analysis was performed to assess the impact of demographic, clinical and procedural characteristics on the study’s endpoint. Variables were assessed in univariate as well as in multivariate Cox proportional model analyses. In the latter, covariates with a threshold of p < 0.10 in the univariate Cox analyses were entered into an initial multivariate model than removed stepwise based on the probability of the likelihood-ratio statistic to determine independent predictors of the clinical endpoint. Improvement over the baseline model was checked with Omnibus tests of model coefficients.

Survival differences between the groups and the cumulative incidence of the clinical endpoint were calculated according to the Kaplan-Meier method. Specificity and sensitivity of platelet function test cut-off points in predicting the occurrence of the primary endpoint were determined by ROC curve analysis. Values of p<0.05 were considered statistically significant and values of p<0.1 were considered as a trend.

Statistical analysis was performed using SPSS Statistics V22 (IBM Corporation, Armonk, NY, USA) and Graph Pad Prism software 5 (GraphPad Software, Inc., La Jolla, CA, USA).

## Results

### Clinical characteristics

Between October 1, 2009 and March 31, 2015, 194 patients (60.6% male) were enrolled in the registry with a median age of 60 (range: 31–86) years. All of the recruited cases were treated during an elective DEB intervention due to stable angina. No patients were lost to follow up. The mean follow-up time was one year. Baseline clinical, procedural, laboratory and treatment characteristics are shown in Table 1.

Based on their cardiovascular risk factors, study patients composed a low-to-moderate risk cohort with 89% hypertension, 49% dyslipidaemia and 25% diabetes. Sixty-nine percent of the patients had previous MI and 19% of them had previous coronary artery bypass grafting (CABG). The vast majority of the patients were treated with clopidogrel (90%) while 9% received prasugrel and 1% of the patients received ticlopidine therapy.

Regarding procedural data, 26% of the ISR cases were found in a previously implanted DES. Eighty-seven percent of the cases underwent predilatation prior to the use of DEBs.

Overall, 152 (78%) patients had ADP-test available after DEB PCI. The reasons for omission of the ADP test were logistic reasons, transfer or discharge of the patient without blood sampling in 14% or unavailable lab on the day after the PCI in 8%. Patients with and without ADP-test available had comparable baseline characteristics except for greater use of allopurinol in those who did not present ADP-test. The median value of the ADP-test after DEB PCI was 28 U.

From the 152 subjects tested, 32 (21%) had HPR according to Multiplate assay. There was a significant difference in the frequency of DES and bare metal stent (BMS) -use by the prior PCI between HPR and no HPR groups; with significantly more DES ISR in the HPR group. In addition, the choice of DEB differed among the groups with or without HPR; however, these parameters did not have an effect on the composite clinical endpoint.

### Clinical outcomes

Thirteen (6.7%) patients had elevated troponin level after the procedure defined according to the universal definition of MI type 4 after the DEB PCI,[9] and other complications occurred in 31 cases (coronary dissection and perforation, no flow) during the DEB PCI (Table 1). Twenty-seven patients reached the composite endpoint during the follow-up period. One patient died due to cardiovascular cause, 12 patients suffered MI during follow-up. Twenty-six patients had a revascularization event, out of that 17 were target vessel revascularisation (TVR). There were no documented cases of stroke (Table 2).

**Table 2:** Clinical outcomes in the patient population and stratified according to the platelet reactivity.

Clinical endpoints	Patient population (n = 194)	no HPR (n = 120)	HPR (n = 32)	HR (95% CI)	p
**Composite endpoint**	27 (13.9)	13 (10.8)	8 (25.0)	2.5 (1.0–5.9)	0.03
**Any revascularization**	26 (13.3)	13 (10.8)	8 (25.0)	2.5 (1.0–5.9)	0.03
**TVR**	17 (8.7)	7(5.8)	5 (15.6)	2.8 (0.9–8.8)	0.06
**MI**	12 (6.2)	6 (5.0)	6 (18.8)	3.9 (1.3–12.2)	0.01
**CV death**	1 (0.5)	0 (0.0)	0 (0.0)	-	-
**TIA/stroke**	0 (0.0)	0 (0.0)	0 (0.0)	-	-

Values are n (%).

HPR = high platelet reactivity; HR = hazard ratio; CI = confidence interval; TVR = target vessel revascularization; MI = myocardial infarction; CV = cardiovascular; TIA = transient ischaemic attack

The rate of the composite clinical endpoint, revascularization and MI were significantly higher in the HPR group compared to patients without HPR ([MACE: HR: 2.5; CI: 1.0–5.9; p = 0.03]; [Revascularisation: HR: 2.5; CI: 1.0–5.9; p = 0.03]; [MI: HR: 3.9; CI: 1.3–12.2; p = 0.01]). Compared with no HPR patients, HPR group showed a non-significant trend for higher rate of TVR (HR: 2.8; CI: 0.9–8.8; p = 0.06) (Table 2).

### Predictors of ischemic events

**Fig 1:**
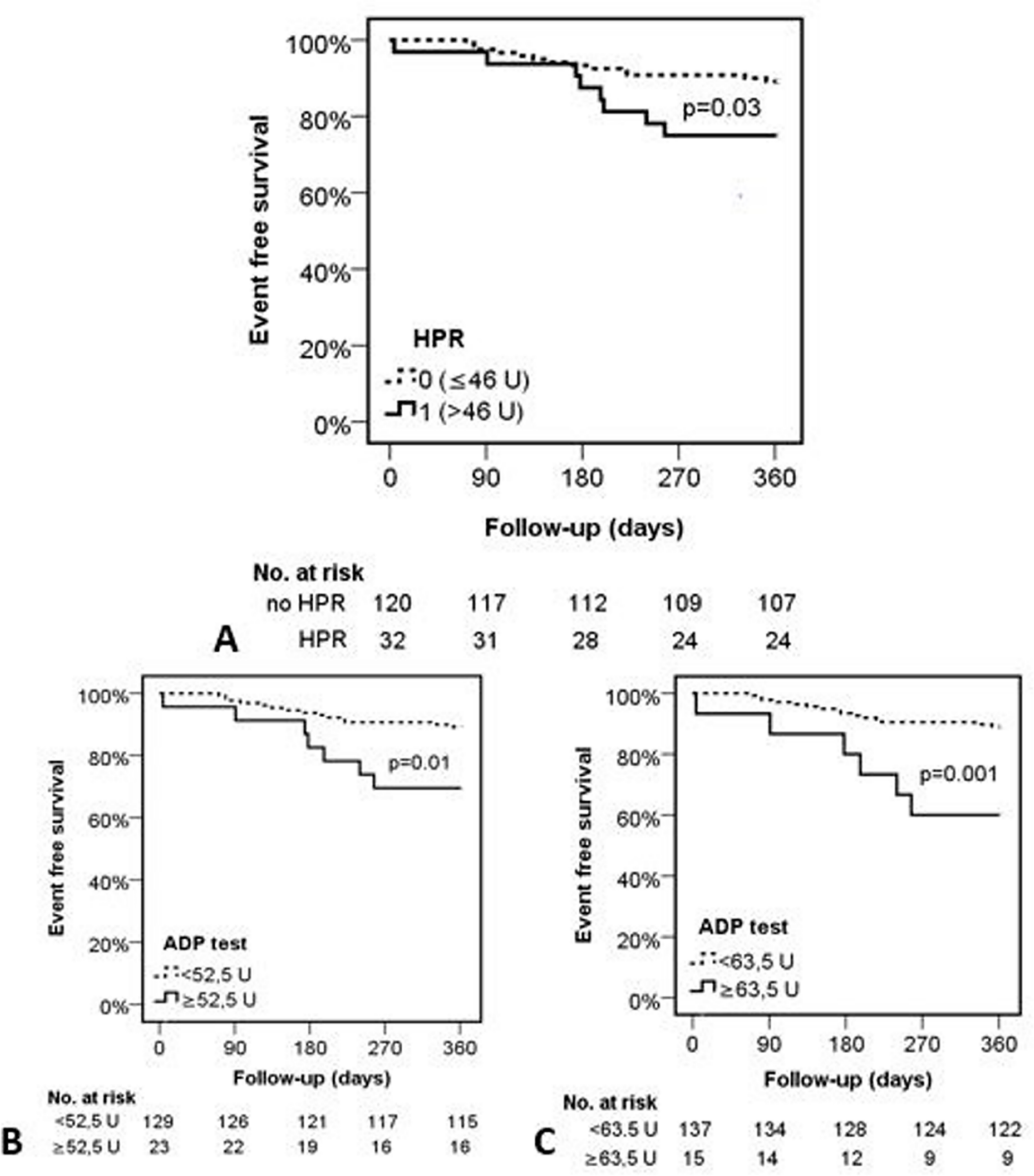
Event free survival of the patients based on the platelet reactivity during the follow up period. **A:** Event free survival of the patients with and without HPR based on the consensus cut-off value. **B, C:** Event free survival of the patients based on the ROC curve analysis identified two potential cut-off values. Event rates at one year are shown for each group as Kaplan-Meier estimates. HPR = high platelet reactivity; ADP = adenosine diphosphate.

According to the Cox regression analyses HPR (HR: 2.45; CI: 1.01–5.92; p = 0.03) (Fig 1A), and prasugrel therapy (HR: 2.74; CI: 1.04–7.26; p = 0.03) were significant predictors of the primary endpoint and only patients with recent myocardial infarction received prasugrel at the time of the DEB procedure.

**Fig 2:**
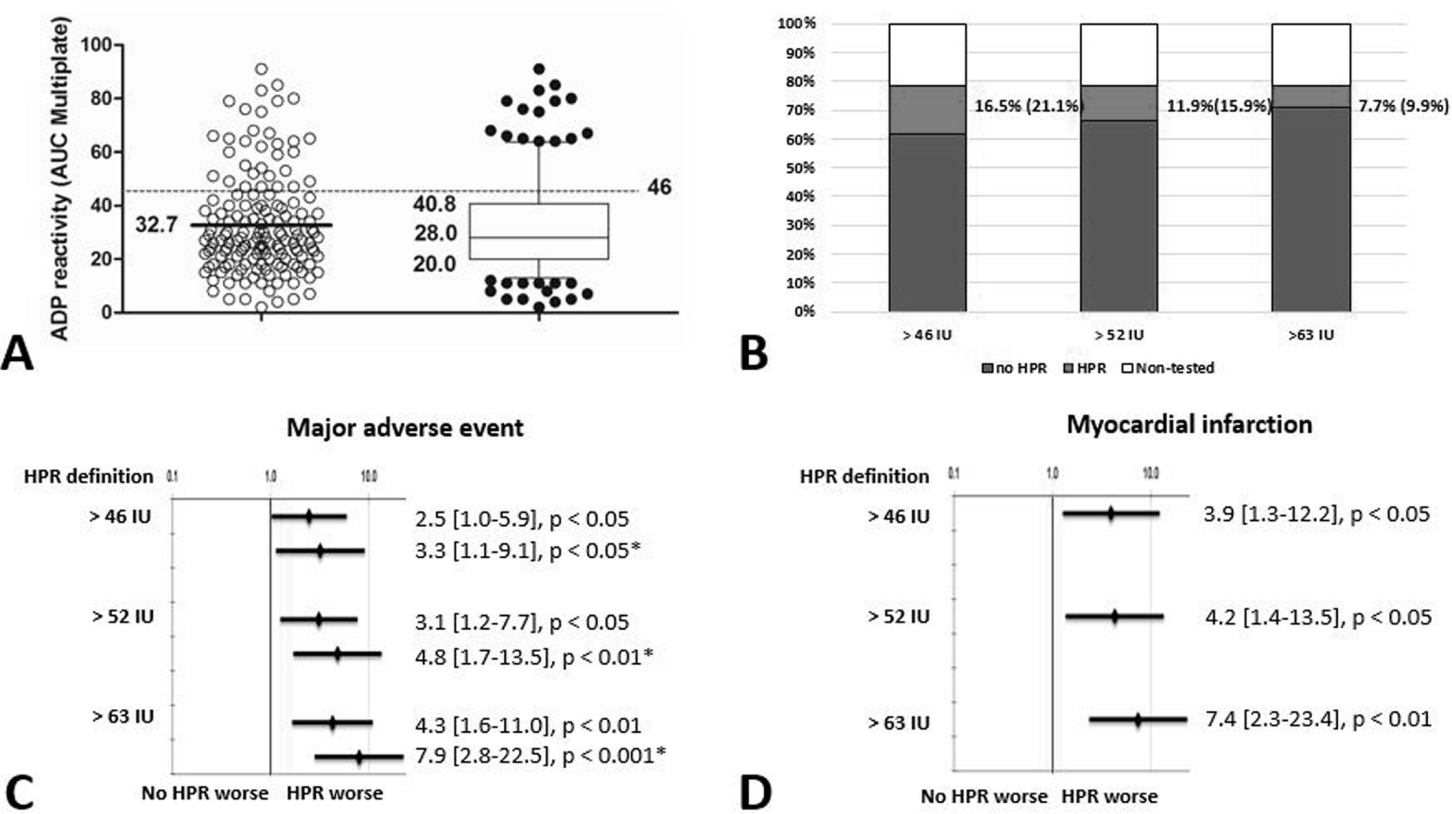
Results of the platelet function test, frequency of high platelet reactivity and its relation to clinical endpoints. **A:** A scatter plot of platelet reactivity with the Multiplate device in all patients. Values are presented as median {25% percentile, 75% percentile}, ADP reactivity presents as U. **B:** Percent of the platelet function tested cases with percent of HPR and no HPR patients in the total cohort based on the platelet reactivity values. **C:** Impact of platelet reactivity on MACE. **D:** Impact of platelet reactivity on MI. Values are presented as HR [95% CI]. *: asterisk marks hazard ratios from multivariate Cox regression analyses. ADP = adenosine diphosphate; AUC = area under the curve; HPR = high platelet reactivity; MACE = major adverse cardiovascular event; MI = myocardial infarction; HR = hazard ratio; CI = confidence interval.

ROC curve analysis identified two potential cut-off values 52.5 U (33% sensitivity, 12% specificity) and 63.5 U (28% sensitivity, 7% specificity) of the platelet function test. Using these and the consensus defined 46 U (38% sensitivity, 12% specificity), Kaplan-Meier analyses demonstrated similarly significant higher risk of composite endpoint ([46 U (HPR): HR: 2.42; CI: 1.01–5.92; p = 0.03]; [52.5 U: HR: 3.09; CI: 1.24–7.67; p = 0.01]; [63.5 U: HR: 4.25; CI: 1.64–10.96; p = 0.001]) with higher risk but smaller at risk population with the higher cut-off values (Fig 1A, 1B and 1C; Fig 2). Based on the Kaplan Meier curve morphology and separation, the consensus cut-off value predicts the risk of later (>60 days) events, whereas, the higher cut-off values are rather predictors for the earlier cardiovascular events (Fig 1A, 1B and 1C).

Furthermore, we found a tendency of poorer outcomes associated with the total length of the DEB (HR 1.02; CI: 0.99–1.05; p = 0.06).

In order to clarify the role of platelet function testing related to other covariates of the primary endpoint multivariate models were generated to identify independent predictors.

According to the multivariate analysis, HPR and the efficacy of ADP receptor antagonist treatment as assessed by the platelet function test remained significant, independent predictor of the primary endpoint ([HPR: HR: 2.88; CI: 1.02–8.14; p = 0.04]; [ADP test, U: HR: 1.03; CI: 1.00–1.05; p = 0.04]) (Table 3). In the multivariate analysis, history of statin treatment and the total length of the DEB were significant, independent predictor of the MACE ([statin: HR: 0.28; CI: 0.09–0.84; p = 0.02]; [total DEB length: HR: 1.04; CI: 1.00–1.08; p = 0.03]) (Table 3).

**Table 3:** Clinical and procedural predictors of MACE at one year.

	Univariate Cox Proportional Hazard Model		Multivariate Cox Proportional Hazard Model	
	HR (95% CI)	p-value	HR (95% CI)	p-value
Age	0.98 (0.95–1.02)	0.56		
Male gender	1.28 (0.57–2.86)	0.53		
Smoking	1.07 (0.44–2.61)	0.87		
Hypertension	2.08 (0.28–15.40)	0.46		
Diabetes mellitus	0.90 (0.36–2.26)	0.83		
Statin treatment	0.49 (0.20–1.17)	0.10	0.28 (0.09–0.84)	0.02
Prior MI	2.20 (0.75–6.41)	0.13		
Prior CABG	1.03 (0.39–2.76)	0.94		
Indication of prior PCI				
• STEMI	1.52 (0.69–3.35)	0.29		
• NSTEMI	0.41 (0.05–3.04)	0.36		
• Unstable angina	0.84 (0.25–2.82)	0.78		
• Stable angina	0.88 (0.39–1.97)	0.77		
Target vessel				
• LAD	0.62 (0.27–1.43)	0.26		
• RCA	1.67 (0.78–3.57)	0.18		
• CX	1.20 (0.41–3.47)	0.73		
• Graft	0.04 (0.00–160.28)	0.26		
Drug eluting stent restenosis	0.76 (0.30–1.88)	0.55		
Type of DEB				
• SeQuent Please	0.63 (0.25–1.58)	0.32		
• Invatec In-Pact	1.39 (0.65–2.97)	0.39		
• Protege	1.42 (0.60–3.37)	0.41		
• Pantera Lux	0.54 (0.12–2.28)	0.39		
Total DEB length, mm	1.02 (0.99–1.05)	**0.06**	1.04 (1.00–1.08)	0.03
Largest DEB diameter, mm	1.01 (0.44–2.32)	0.97		
DEB count/patient	1.52 (0.75–3.05)	0.24		
Predilatation	1.24 (0.37–4.12)	0.72		
ADP-test, U	1.02 (0.99–1.04)	**0.06**	1.03 (1.00–1.05)	0.04
Prasugrel treatment	2.74 (1.04–7.26)	0.03		

HR = hazard ratio; CI = confidence interval; MI = myocardial infarction; CABG = coronary artery bypass graft; PCI = percutaneous coronary intervention; STEMI = ST-segment elevation myocardial infarction; NSTEMI = non–ST-segment elevation myocardial infarction; LAD = left anterior descending; RCA = right coronary artery; Cx = circumflexus; DEB = drug eluting balloon; ADP = adenosine diphosphate; MACE = major adverse cardiovascular event

## Discussion

The main finding of our study is that HPR may be a predictor of adverse ischemic events in stable angina patients treated with DEB due to ISR. The higher rate of ischemic events was predominantly triggered by a higher risk for MI and repeat revascularization. In addition to HPR, total DEB length and statin treatment were shown to significantly interfere with clinical outcomes.

Several randomized studies demonstrated the safety and efficacy of DEB for the treatment of ISR.[3] [4] Based on the results of the PACCOCATH ISR [12] [3] and PEPCAD II [13] trials, the European Society of Cardiology/European Association for Cardio-Thoracic Surgery guidelines for coronary revascularization gave a class IIa recommendation for this treatment modality.[4] Previous registries and studies investigated the correlation between patient and procedural characteristics and clinical outcome with heterogeneous results. Our cohort comprised a routine all-comer population with low-to-moderate risk clinical and procedural features. The incidence of the composite end point was 14% during the 1-year follow-up period, higher than in randomized trials (9% in PACCOCATH ISR I and II and in PEPCAD II),[3] [13] but similar to a multicentre prospective registry.[14] Regarding to procedural characteristics, DEB length was found to be an important predictor of adverse outcomes: the longer the DEB, the higher the risk of ischemic events. Although the length of DEB should be selected to fully cover the restenotic segment, operators should find the shortest appropriate size, without large mismatch.

After DEB PCI, patients receive dual antiplatelet therapy but the optimal intensity and length of the therapy in this patient group is not fully defined. Clopidogrel non-responsiveness and HPR is a strong independent predictor of recurrent ischemic events and mortality after coronary stent implantation [5] and may play a relevant prognostic role in patients after DEB PCI. This association is strongest in patients with ACS, likewise, more potent P2Y12-inhibitors are used to prevent thrombotic recurrences.[15]

Earlier OCT examination discovered uncovered or malapposed stent struts immediately after the DEB procedure and in all images dissections were seen throughout the DEB-dilated segment which were not visible with angiography and remained untreated.[6] Therefore, although DEB PCI was shown to be an effective treatment for ISR, it may result in a large prothrombotic surface with delayed healing consequent to the paclitaxel treatment. This represents a potential risk and may necessitate effective antiplatelet therapy to prevent adverse events.

In line with the intraluminal imaging findings, we found a significant association between platelet reactivity and adverse outcomes and patients with HPR had a 2.5-fold higher risk for ischemic events. The higher risk was mainly driven by MI and revascularization, while stent thrombosis (ST) and mortality were rare.

This is in line with earlier randomized trials and a multicentre registry showing a low rate of early thrombosis of the DEB treated stented segment. [14] [16] [17] In our study, there was only one diagnosed ST (0.5%) after 4 days of the procedure, in a patient with HPR.

In patients with HPR most of the repeated revascularisations were triggered by events of acute MI (75% of revascularisation and 80% of TVR), whereas, in patients without HPR 46% of revascularisations and 57% of TVR were performed because of an acute MI. In-stent restenosis can frequently present as MI [18][19][20] and angiographically, patients with MI tend to have an aggressive pattern of restenosis and total occlusion of the target lesion. One of the most likely explanation of MI in ISR include late stent or device thrombosis, which can be caused by incomplete neointimal coverage, early termination of antiplatelet therapy and/or increased neointimal thrombogenic tissue factors such as tissue factor and collagen.[18] The average time between the DEB procedure and the appearance of adverse events was 6 months (mean 181 days) in our study. When assessed with OCT at 6 months >94%, therefore almost complete neointimal coverage was found after stent implantation postdilated with DEB [21]. Based on these findings, incomplete neointimal coverage may play a less important role in the mechanisms of late ischemic events also but supports the relevant role and importance of ineffective antiplatelet therapy and residual platelet reactivity in the mechanisms of late ST and occurrence of repeated MI.

As a consequence of paucity of relevant data corrective treatment in terms of intensification of antiplatelet therapy based on platelet function studies is not established. Currently available large scale, randomized trials showed the prognostic role of HPR in patients underwent coronary stent implantation but failed to demonstrate the clinical improvements when treatment modifications were implemented on the basis of platelet function testing in patients with elective PCI.[22] [23] [24] [25] The TRIGGER-PCI trial testing switching from clopidogrel to prasugrel in patients with HPR found the 6-month event rate after DES implantation extremely low and the study stopped prematurely due to futility. [23] In two further trials, in the GRAVITAS and ARCTIC trials, treatment modification that mainly consisted use of high-dose clopidogrel in patients with high platelet reactivity after elective PCI with DES did not reduce the rate of end points compared with standard therapy.[22] [24] Using different primary end-point definition, we found a 14% event rate in our real-life cohort and a significantly greater rate of the composite end-point in patients with HPR compared to patients without HPR (25% vs 11%) after PCI with DEB while there were no significant differences in clinical, laboratory and treatment parameters between the HPR and NPR group. Patients treated with prasugrel because of an acute coronary event within one year had worse outcome in our study. As this difference persisted in multivariate analyses taking antiplatelet efficacy in account we hypothesize that this worse prognosis is rather explainable with the recent ACS than with the antiplatelet therapy itself. Furthermore, due to the low numbers and lack of randomized comparisons and protocolled treatment modification our data do not allow drawing conclusion regarding the efficacy of corrective treatment. According to the multivariate analysis, beside platelet reactivity, history of statin treatment and the total length of the DEB were significant, independent predictors of the cardiovascular events. The other clinical and procedural characteristics had no important influence on the outcomes. This finding is in contrast with the earlier published registry from Calé et al. In their analysis of 156 patients the predictors of poorer outcome were previous MI and CABG, acute coronary syndrome at presentation, and PCI in the LAD, while DEB length and dyslipidaemia were not predictive of one-year outcome.[14] In our study, only elective DEB treated ISR patients were recruited which cohort is dissimilar to the populations of Calé et al. with acute coronary syndrome and small vessel disease included which may explain the differences in the verified determinants of worse results.

Our analyses of the predictive value of different level residual platelet reactivity identified two potential alternative cut-off values. Using these and the consensus defined 46 U, Kaplan-Meier analyses demonstrated similarly significant higher risk of composite endpoint with higher risk but smaller at risk population with the higher cut-off values (Fig 1 A, 1B and 1C; Fig 2). Different time-distribution of end-points and separation patterns of the Kaplan Meier were observed using these values. Using the lowest consensus cut-off value, we observed a late (>60 days) occurring difference of event frequencies, whereas the higher cut-off values appeared to be better predictors for the earlier events. These findings may draw the attention to the fact that the proposed cut-off values for platelet function tests are mainly based on stent implanted ACS populations while in different clinical scenarios the predictive value and the optimal cut-offs may differ.

### Study limitations

We have to acknowledge some limitations of our registry. The most important limitations are the single-centre design and consequent small sample size and the lack of blinding. It was left to the discretion of the operator to perform platelet function testing and not all of the patients were tested. Furthermore, although our inclusion criteria allowed the inclusion of ISR patients also presenting with acute coronary syndrome all of the cases entered the registry were treated during an elective intervention. This allows only to draw conclusions regarding patients with elective DEB treatment.

## Conclusion

In stable angina patients treated with DEB due to ISR, HPR is significantly associated with a higher risk for recurrent ischemic events, mostly due to a higher risk for MI and revascularization. Regarding procedural characteristics, DEB length was an independent predictor of worse outcome. Further studies may investigate the safety and efficacy of treatment modification regarding P2Y12-inhibitor therapy and the optimal duration of treatment in DEB patients with HPR.
